# Research on strain aging behavior of ultra-high strength dual-phase steel

**DOI:** 10.1016/j.heliyon.2023.e20960

**Published:** 2023-10-13

**Authors:** Biao Xiao, Jie Zhou, Jean-Luc Christen, Weimin Zeng, Wenyi Peng

**Affiliations:** a School of Physics and Materials Science, Nanchang University, Nanchang, China; b Valin ArcelorMittal Automotive Steel Co., LTD., Loudi, China; c ArcelorMittal Global R&D, Maizières-lès-Metz, France; d School of Materials and Metallurgy, Wuhan University of Science and Technology, Hubei, China

**Keywords:** Strain aging, Dislocation pinning, Bake hardening value, Dual-phase steel, Upper yield stress, Tensile specimen

## Abstract

The bake hardening value is one of the vital strength indexes of dual-phase steel, representing the strengthening ability of materials after pre-strain and baking, playing an important role in vehicle safety and lightweight design. Studying and improving the strain aging mechanism of dual-phase steel helps one to understand the material characteristics and enhances its utilization value. However, the ultra-high strength dual-phase steel is often prone to fracture outside the gauge length of a tensile specimen of the bake hardening value test. No suitable theory explains the fundamental law of dislocation pinning during the saturation stage at present. This paper used FEA, DIC, SEM, TEM, internal friction, and metallographic methods to study the strain aging behavior of dual-phase steels under different pre-strain, bake time, and bake temperature conditions. The results show that the fracture outside the gauge length is related to factors such as the uneven distribution of pre-strain and the ultra-high upper yield strength. The rolling pin shape tensile specimen testing has successfully solved this testing problem. The measured results at the saturation stage of dislocation pinning are in good agreement with the fitting results of the dislocation pinning strengthen mechanism based on the probability event quantization assumption.

## 1 Introduction and theoretical background

Usually, strain aging is classified into dynamic and static strain aging. Dynamic strain aging may appear at a sufficiently higher temperature or low strain rate of some materials. It is caused by repeated locking and unlocking of dislocation during deformation, leading to serrations on the tensile curve, known as the Portevin-Le Chatelier effect [1]. Because of the dislocation locking in steel by interstitial atoms, there will be a static strain aging phenomenon where the stress-strain curve after reloading does not follow the original curve before reloading [2,3]. Except for some materials, such as interstitial free steel, in which interstitial carbon and nitrogen atoms were scavenged by titanium or niobium to achieve non-aging and no yield point phenomenon [4], the static strain aging phenomenon could be found in a variety of materials, including low carbon steel [5,6], low-alloy steel [7], dual phase steel [8], duplex stainless steel [9]. This phenomenon even exists in α-titanium [10], titanium aluminum alloys [11], NiAl-based alloys [12], and many metal materials. The topic discussed in this paper mainly focuses on static strain aging.

Chemical compositions of dual-phase steel have essential effects on aging behavior. The aging in dual-phase micro-alloyed steel occurs more slowly than the dual-phase carbon steel because of nitrogen and carbide formation by alloys [13]. The effect of the bake hardening index decreased by alloy is also observed on low-carbon steel [14]. The increase in Si% from 0.34 % to 1.51 % for the dual-phase silicon steels increased the bake hardening (BH) value. However, the BH value was decreased to a negative value as Si% increased to 2.26 %. The decrease in dual-phase silicon steels is attributed to the decomposition of martensite and releasing of internal stresses [15].

The heat treatment process of materials, aging time, pre-strain value, aging temperature, strain path, grain size, and many other process factors have insignificantly affected aging behaviors [16]. Increasing the inter-critical annealing temperature for low-carbon dual-phase steel decreases solute carbon in the ferrite phase. After quenching, the natural quench aging effect will be reduced [17]. In a lower range of pre-strain, the higher BH values are achieved with higher pre-strains. The further pre-straining does not improve the BH value but presents a tendency for a decline in BH value [18]. The commercial dual-phase steel grade DP600 and transformation induced plasticity grade TRIP700 both have similar bake hardening behavior. Take cross-section reduction due to pre-strain, the strengthening peak is reached with lower pre-strains of 2 % for DP600 and 5 % for the TRIP700 steel [19]. Due to different initiation micro-crack growing behaviors, the DP600 produced by the continuous annealing process shows a maximum 70 MPa BH value at 2 % pre-strain. Differently, the DP600 produced by continuous heating annealing was observed to have a delayed maximum 72 MPa BH value at 4 % pre-strain [20].

A two-step yield strength increase during strain aging of dual-phase steel is observed, namely the first plateau due to Cottrell atmosphere formation and the second plateau due to precipitates formation. Subsequently, an over-aging stage decreases yield strength [8]. Interestingly, the research on bainite-strengthened complex-phase steel shows that the over-aging stage produces cementite and increases yield strength [21].

Also, microstructures of higher volume fraction of martensite, smaller martensite islands, and smaller ferrite grains can produce higher BH values [18]. Under constant strain-aging parameters, the martensite volume fraction between 0.22 and 0.39 has an insignificant effect on the strengthening due to the bake hardening effect. But decreasing the martensite fraction to 0.17 and increasing it to 0.86 reduces the bake hardening response of the second strengthening stage due to carbide precipitation [22]. For the ultra-low carbon bake hardening steel, the degree of atmosphere formation at the saturation stage is independent of the different pre-strain values [6,23].

The BH value has been seen as the strengthened contributions of Cottrell atmosphere formation, the formation of carbides, and the tempering of martensite [24]. The effect of stress relief due to martensite tempering should also be considered [15,25]. Regarding the Cottrell atmosphere formation stage, the Cottrell-Bilby theory explains that dislocations, particularly for edge dislocation, could be locked by some smaller interstitial solute atoms, such as carbon and nitrogen, which gather around the dislocations and form a so-called Cottrell atmosphere [26]. When the dislocations break away from the Cottrell atmosphere by stress, yielding occurs. As a result, the Lüders bands may be produced during deformation [27]. Cottrell and Bilby described the atmosphere formation till saturation, with the degree of atmosphere formation at the time t is given by,

(1) $$\frac{N_{t}}{N_{s}}=3n_{0}\lambda {\left(\frac{\pi }{2}\right)}^{\frac{1}{3}}{\left(\frac{ADt}{kT}\right)}^{\frac{2}{3}}$$

where $N_{t}$ is the number of the carbon atoms which arrive within the time t; $N_{s}$ is the total number of carbon atoms per unit length of the dislocation required to form an atmosphere of the one atom per atom plane; $n_{0}$ is the average concentration of carbon atoms per unit volume; λ is the slip distance in the dislocation; A is interaction parameter related to rigidity modulus, Poisson's ratio, dislocation slip distance and volume change caused by carbon, etc.; D is the carbon diffusion coefficient; k is the Boltzmann constant; T is the temperature in K.

Hartley considered $\Delta \sigma /\bar{\sigma }$ as a linear function of $t^{2/3}$ with the slop S related to the diffusion coefficient of carbon [28]. The increase in strength Δσ is defined as the difference of upper yield strength $R_{eH}$ after strain aging at time t and the flow stress $\sigma_{x}$ at the end of pre-straining. The value of $\bar{\sigma }$ is equal to $1/2\left(\sigma_{x}+R_{eH}\right)$. The slop S is given by,

(2) $$S=k{\left(\frac{D}{T}\right)}^{\frac{2}{3}}$$

where $D=D_{0}e^{-1\Delta H/RT}$; ΔH is the enthalpy of activation; $D_{0}$ is a constant that should be measured [29].

The yield strength of the steel was estimated with a linear combination of the strengthening mechanisms calculated by $\sigma_{y}=\sigma_{0}+\sigma_{ss}+\sigma_{p}+\sigma_{GB}+\sigma_{dis}$. where the $\sigma_{0}$ is friction stress; $\sigma_{ss}$ is solid solution strengthening; $\sigma_{p}$ is the particle strengthening; $\sigma_{GB}$ is grain boundary strengthening, and $\sigma_{dis}$ is dislocation strengthening [30]. Generally, twin boundaries also act as an obstacle to the movement of mobile dislocations. Thus, the twinning strengthening $\sigma_{T}$ is a contribution value of the strengthening mechanisms [31]. Then, the linear function will be written as $\sigma_{y}=\sigma_{0}+\sigma_{ss}+\sigma_{p}+\sigma_{GB}+\sigma_{dis}+\sigma_{T}$.

The fraction stress $\sigma_{0}$ will be affected by alloys in the materials. It's usually considered 45 MPa in pure iron [32]. The increase in alloy element content results in higher fraction stress, but does not simply increase the strength [33]. The solid solution strengthening $\sigma_{ss}$ was contributed by different alloy elements, including substitutional and interstitial elements [34]. The particle strengthening was expressed by the Ashby-Orowan equation. As to the types of precipitations, it should note that there are the co-clustering, co-precipitation, and cluster-precipitation reactions, their interactions, and resulting individual or combined strengthening. The clustering and precipitation in different phases, including ferrite, martensite, bainite, etc., affect the strengthening behaviors [35]. Generally, the grain boundary strengthening is described by the Hall-Petch relationship. The square root of grain sizes of different phases in the material was usually considered [36].

The hardening laws developed by Taylor in 1943 [37] show that the total dislocation strengthening is proportional to the square root of dislocation density $\sqrt{\rho }$. The yield stress will follow the relationship,

(3) $$\sigma_{y}=\sigma_{0}+M_{T}\alpha \mu b\sqrt{\rho }$$

$\sigma_{0}$ is the strengthen term related to friction, solid solution, particle, grain boundary strengthening, and so on; $\rho_{f}$ is the dislocation density; $M_{T}$ is Taylor factor; α is dislocation type and distribution factor; μ is shear modulus; b is the magnitude of Burgers vector.

Research shows [38] that the fraction of relatively immobile dislocation or forest dislocation density accounted for the full nonadditive strengthening. This strengthening model of yield stress $\sigma_{y}$ is expressed as:

(4) $$\sigma_{y}=\sigma_{0}+\left(\sigma_{ss}+\sigma_{p}+\sigma_{GB}\right)\left(1-\frac{\rho_{f}}{\rho_{m}+\rho_{f}}\right)+M_{T}\alpha \mu b\sqrt{\rho_{f}}$$

In the formula above, $\rho_{m}$ is the mobile dislocation density; $\rho_{f}$ is the relatively immobile or forest dislocation density.

Meanwhile, an investigation of the strain rate and dislocation density dependence of the strength presented [39] shows that when the yield dislocation density $\rho_{y}$ is lower than a critical dislocation density $\rho_{c}$, the yield stress decreases with increasing dislocation density. For $\rho_{y}>\rho_{c}$, the yield stress increases with increasing density, responding to the strain rate-independent.

After being delivered to automotive customers, the dual-phase steel (DP steel) will be produced into parts with stamping, painting, and baking [40]. During the stamping process, dislocations increment will promote to form forest of dislocations, leading to an increase in yield strength. Moreover, the baking process of the part provides additional energy for interstitial C and N atoms to move to the dislocations for pinning and form a Cottrell atmosphere, further improving the yield strength of the part. The strain aging strengthening effect of dual-phase steels can be widely used to enhance the structural strength of parts, which plays a vital role in vehicle safety and lightweight design [16].

The BH value is a crucial strength index of dual-phase steel, representing the strengthening ability under a particular strain aging condition. This strengthening ability of ultra-high strength dual-phase steel is particularly prominent, and its BH value can exceed 150 MPa [18]. In the first stage, in the beginning, the strain aging behavior obeys Cottrell–Bilby's theory very well. Regarding the saturation stage, Cottrell–Bilby's view is limited in explaining how the strain aging behavior goes on. Further research on the strain aging behavior and strengthening mechanism of ultra-high strength dual-phase steel help understand the characteristics of the material and improve its utilization value. However, in the test of BH values for ultra-high strength dual-phase steels, fractures often occur outside the gauge length of the tensile specimens, affecting measurement accuracy. It is not even possible to identify the yield strength on the tensile curve. Therefore, there is an urgent need to find a solution for the BH value test of ultra-high strength dual-phase steel.

This paper analyzed the reason for fracture outside gauge length of a tensile specimen of bake hardening value test. The testing problem has been successfully solved by a rolling pin shape tensile specimen testing method. This paper used FEA, DIC, SEM, TEM, internal friction, and metallographic techniques to study the strain aging behavior of dual-phase steels under different pre-strain, bake time, and bake temperature conditions. The dislocation pinning strengthen mechanism based on quantization assumption and probability event can explain the fundamental law of dislocation pinning saturation stage.

## 2 Materials and experimental details

### 2.1 Materials

**Fig. 1:**
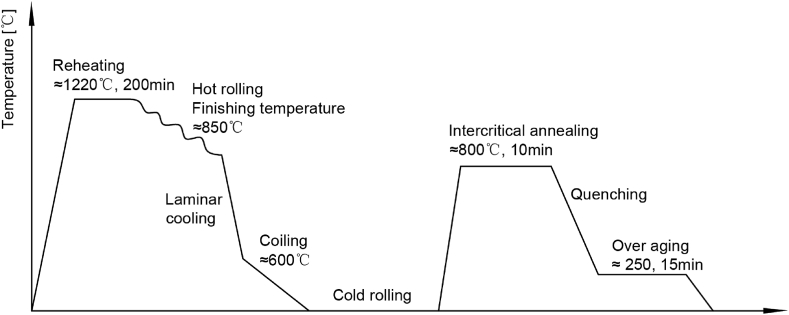
Hot rolling, cold rolling, and annealing process route for DP1 to DP4.

Four DP grades, named from DP1 to DP4, are researched in this paper with different compositions, as shown in Table 1. The tensile strength of ultra-high strength dual-phase steel DP4 is higher than 1200 MPa. The BH value of DP4 is much higher than other grades. Some special aging behaviors on DP4 were noticed. This paper mainly focuses on the ultra-high strength DP4. Steelmaking was completed in a plant of the steel company. The slabs were cast in continuous caster. Hot rolling was implemented with reversible roughing rolling and continuous finishing rolling. Hot rolled coils were rolled into a full hard strip on the continuous cold rolling mill after pickling. DP steels' process routes are the same, shown in Fig. 1.

**Table 1:** Chemical composition of materials.

Material	Composition, %					
	C	Si	Mn	P	S	B
**DP1**	0.09	0.31	1.59	0.0012	0.0008	0.0002
**DP2**	0.12	0.32	1.60	0.0013	0.0010	0.0004
**DP3**	0.09	0.29	2.61	0.0011	0.0010	0.0028
**DP4**	0.12	0.30	2.60	0.0012	0.0007	0.0029

The cold rolled strip was annealed in a plant with continuous annealing lines. Then, the strips were quenched by blowing boxes with air subsequently. After cooling, strips were treated in the over-aging furnace to adjust the martensite state and avoid aging problems when using [41]. The inter-critical annealing time was about 10 min. The over-aging time was about 15 min. In the cooling section, the cooling rate was higher than 30 °C per second to produce martensite. The annealing process conforms to the industrial production condition as researched.

### 2.2 Test methods and equipment

The materials were produced to the same thickness of 1.2 mm. The samples for research were taken from 1/4 of the width of the strip. Subsequently, the specimens for tensile test, microstructure analysis, internal friction analysis, etc., were produced by wire-electrode cutting.

#### 2.2.1 Tensile test

**Fig. 2:**
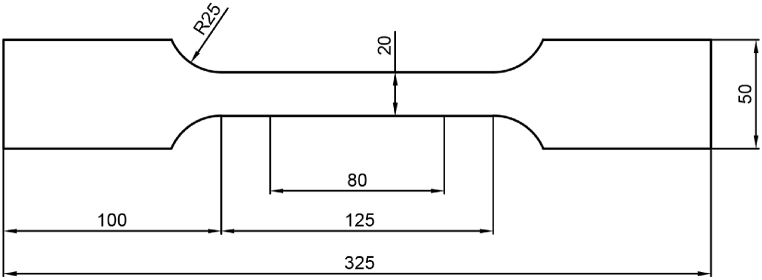
Type 1 geometry for tensile testing (dimensions are given in mm).

The geometries of tensile specimens used in this paper include Type 1 in Table B1 per ISO 6892–1:2019(E) (Fig. 2) and a recommended rolling pin shape tensile specimen in this paper (Fig. 7). The BH value testing process was carried out per BS EN 10325:2006. In other words, specimens were treated with 2 % pre-strain and baked at 170 °C for 20 min. For aging research, specimens were treated with 1.0 %–2.5 % pre-strain and different baking parameters. Instead of the Type 1 specimen, the rolling pin shape tensile specimen was used to test ultra-high strength DP steel during pre-strain to avoid fracture out of gauge length. Tensile tests were carried out by an automatic test system (Zwick Z150 roboTest L) at constant room temperature. The measurement range of the tensile test system is 300 N to 150 KN. During the pre-strain process and tensile testing after baking, the stress rate of the modulus of elasticity testing was 30 MPa/s, the strain rate of yielding was 0.00025 $s^{-1}$, the strain rate after yielding was 0.0067 $s^{-1}$. The strain and stress rates were kept within a relative tolerance of ±20 % as EN ISO 6892–1:2009 (E) required.

#### 2.2.2 Fracture surface analysis by SEM

Scanning electron microscopy (SEM, Zeiss EVO18) was employed to observe the fracture surface of the tensile test specimen. The extra high tension (EHT) was set as 20 kV. The secondary electrons were used for morphology analysis. The working distance (WD) was set as 8.5 mm. The fracture samples for SEM analysis were cut from a fractured tensile specimen. Fracture samples were cleaned with compressed air and fixed on the SEM sample table with a metal clip.

#### 2.2.3 Metallography analysis by microscope

The metallography method with a microscope (Zeiss Axio Observer Z1m) was employed for the ferrite and martensite observation. Samples for analysis were cut into 10 mm by 10 mm and inlaid with resin for surface observation. The surfaces of the samples were polished with sandpapers and finished with 1 μm SiC. Then samples were corroded with 4 % nitric acid (volume fraction) for seconds. The percentages of martensite and ferrite were calculated by Image-Pro Plus on the metallographic photos after grayscale and contrast adjusting.

#### 2.2.4 Austenite observation by XRD

The X-ray diffraction (XRD) system (X'Pert³ MRD) was employed for the austenite observation. The software Jade and Image Pro Plus were used for phase percentage calculation. The XRD patterns of materials at room temperature were tested by Co Kα radiation. The step size was 0.05*°*, while the 2θ results in the range of 40*°*–130*°* were measured with the test speed of 2*°* per minute. Samples for analysis were cut into 10 mm by 10 mm. The surfaces of the samples were polished with 1 μm SiC.

#### 2.2.5 Precipitation analysis by FESEM

A field emission scanning electron microscope (FESEM, ZEISS Sigma 300) was used for nanoprecipitation analysis. The extra high tension (EHT) was set as 5 kV. The InLens detector was used for morphology analysis. The working distance (WD) was set as 5.4 mm. Samples for analysis were cut into 10 mm by 10 mm and inlaid with conductive resin for cross-section observation. The inlaid samples were polished with 1 μm SiC and then corroded with 4 % nitric acid (volume fraction) for seconds.

#### 2.2.6 Dislocations analysis by TEM

Transmission electron microscopy (TEM, FEI Talos F200) was employed to examine the dislocations in the steel with 2 % and without pre-strain. Wet sandpapers were first used to reduce the samples' thickness for TEM analysis. Then, the samples' thickness should be carefully polished into foil with a thickness lower than 100 μm. It must be ensured that no bending or deformation was applied on the foil during polishing. The samples were punched into Φ3mm foil cycles. The thin foil cycle samples were prepared by the method of ion-beam thinning by the Precision Ion Polishing System (PIPS, Gatan 691). Then the dislocations and their interfaces of the thin foil samples were studied by TEM.

#### 2.2.7 Temperature-dependent internal friction measurements

Internal friction research was conducted on a multifunction internal friction apparatus (MFIFA) to study the internal defects interaction and bake-hardening mechanism. Specimens with a dimension of 1.2 mm by 1.5 mm by 45 mm were cut from the samples above for damping-capacity measurement. The surface roughness of the specimen was polished with SiC to lower than 0.1 μm. The internal friction test temperature ranged from room temperature to 380 °C. The heating rate was 2 °C/s.

#### 2.2.8 Strain distribution measurement by DIC

A digital image correlation (DIC, XTDIC-CONST-SD) system captured the strain distribution on the tensile specimens. The DIC system provided non-contact strain data for tensile testing on specimens. The displacements were measured at the subset of the pixels within the area selected, and the full-field strain was computed with tensor options. The displacement measurement accuracy was 0.01 pixels. The pixels of the camera were 5 million. The strain measurement accuracy was ≤20με. The tensile test speed was set as 0.5 mm/min.

#### 2.2.9 Finite element analysis

MSC.Marc is an advanced nonlinear finite element analysis software that can handle various linear and nonlinear structural analyses. It provides a rich library of structure, continuous, and special elements. It was employed for tensile test simulation analysis in this paper. The structural analyses part of the MSC.Marc was used for tensile test analysis to calculate von Mises stress, shear stress, equivalent plastic strain etc., on the tensile specimen.

#### 2.2.10 Oven used for baking and heat treatment

For the BH value testing, the specimens shall be stretched with 2 % pre-strain at the first step and then baked at 170 °C for 20 min. For the aging trial of materials, the sample shall be baked at 70 °C–360 °C for 2–15000 min. The thermostatic oven with an accuracy of ±1.5 °C (YAMATO DF411C) was used for sample heat treatment.

## 3 Results

### 3.1 Tensile testing

#### 3.1.1 Standard tensile testing

The mechanical properties of as-delivery materials without pre-strain and baking are given in Table 2. The materials were produced with the same process shown in Fig. 1, but different strengths have been gained. From the material DP1 to DP4, the higher the strength, the lower the elongation as expected.

**Table 2:** Mechanical properties.

Material	Sample No.	Yield strength, MPa	Tensile strength, MPa	The total elongation, %
DP1	1	384	664	26.0
	2	386	664	25.5
	3	380	668	26.0
DP2	1	436	792	18.5
	2	440	794	21.5
	3	437	795	20.0
DP3	1	738	1040	13.0
	2	747	1044	11.5
	3	749	1039	12.5
DP4	1	924	1265	9.0
	2	921	1270	9.0
	3	921	1273	9.0

**Fig. 3:**
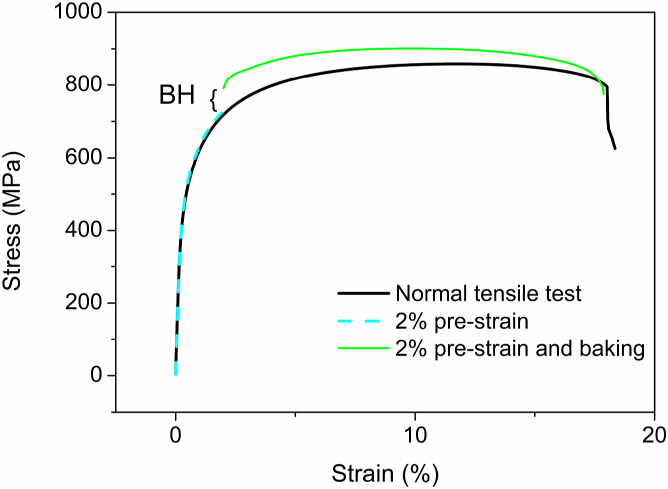
Tensile curves of normal tensile test, 2 % pre-strain and 2 % pre-strain baked in 170 °C baked for 20 min. The bake hardening value is the increment of yield strength after baking.

For the standard baking hardening testing (SBH) per BS EN 10325:2006, the specimen was stretched with 2 % pre-strain at the first step and then was baked at 170 °C for 20 min. Right after baking, the second tensile test will be implemented to calculate the BH value shown in Fig. 3.

**Fig. 4:**
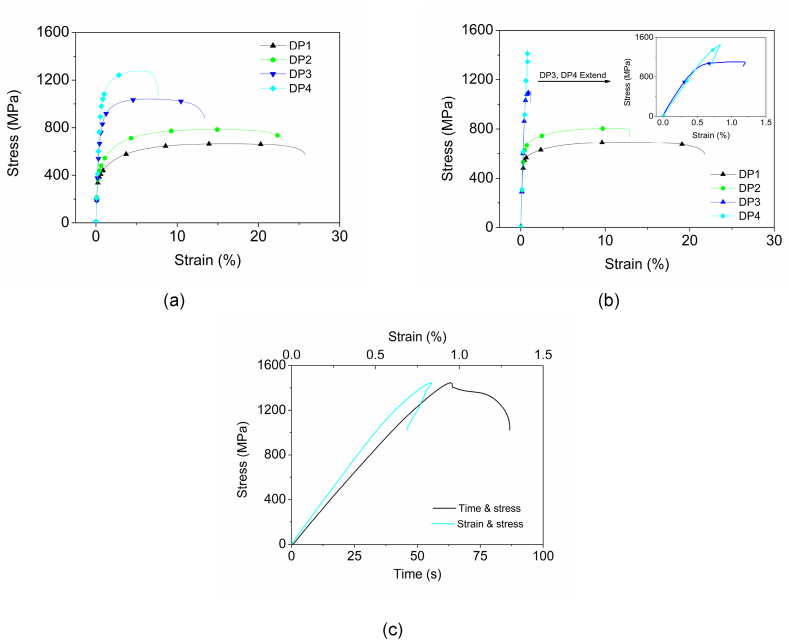
Tensile curves with and without baking. (a) Tensile curves of specimens as-delivery without pre-strain and baking. (b) Stress and strain curves of DP1, DP2, DP3, and DP4 with 2 % pre-strain and baking. (c) Stress and time compared with Stress and strain curve of DP4 with 2 % pre-strain and baking.

**Fig. 5:**
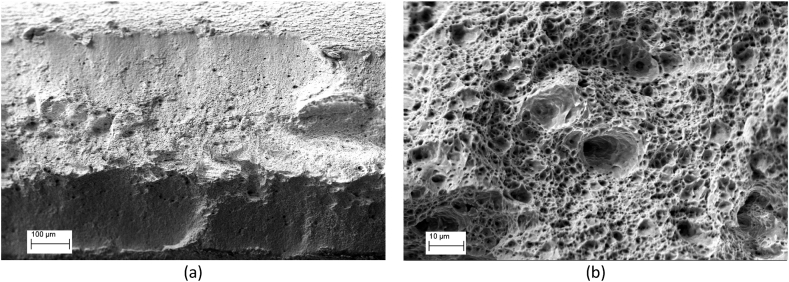
Morphology analysis by secondary electrons on the fracture surface of DP4.

Fig. 4 (a) shows that no yield point elongation can be seen on the tensile curves without pre-strain and baking. For the specimens with 2 % pre-strain and baking, no yielding occurs on DP1 and DP2 Fig. 4 (b), but abnormal curves of DP3 and DP4 in Fig. 4 (b) with a retraction on the tensile curve tail were presented as shown in the extended figure. Morphology analysis by secondary electrons on the fracture surface of DP4 shows a plastic dimple characteristic (Fig. 5). Compared with the strain and stress curve, the stress and time curve of DP4 in Fig. 4 (c) shows that the lower-yield strength could be at the intersection of the slope and yielding platform, which is approximately 1403 MPa.

#### 3.1.2 Rolling pin shape tensile specimen test

**Fig. 6:**
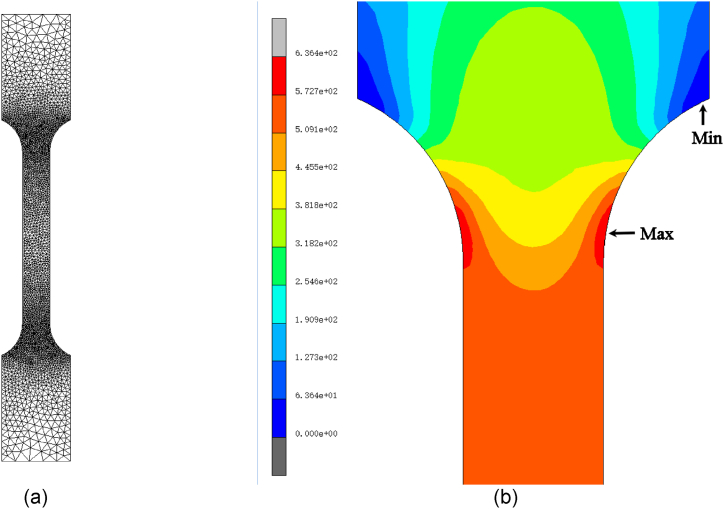
Finite element analysis for the tensile specimen. (a) Mesh generation of finite elements. (b) Von Mises stress distribution shows the stress concentration on the shoulder.

The finite element analysis method implemented a numerical simulation of the stress state for tensile specimens. The Delaunay method was used for six-node element automesh division with applied curve divisions of pre-automesh. There were arranged higher density of elements on the shoulder of the specimen for accurate results, as Fig. 6 (a) shows. The radius of the shoulder on the specimen was 25 mm. The elastic-plastic isotropic type was used for the material. Yong's module was 210 GPa. The tensile curve of DP4 was input into the table database and used for the material plasticity properties. One side clamp section of the specimen was fixed. The other side clamp section applied a boundary condition of a tensile speed of 0.033 mm/s.

**Fig. 7:**
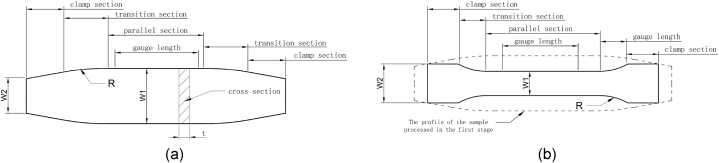
Rolling pin shape tensile specimen. (a) Tensile specimen of the first stage. (b) Tensile specimen of the second stage.

The conclusion of stress concentration on the shoulder was obtained from the analysis, as shown in Fig. 6 (b). The nodal stress was used to calculate the stress concentration factor, $K_{f}$ [42].

(5) $$K_{f}=\frac{\sigma_{\max}}{\sigma_{nominal}}$$

where $\sigma_{\max}$ is the maximum nodal von Mises stress; $\sigma_{nominal}$ is the average von Mises stress in the gauge section. When the radius of the shoulder arc was 25 mm, the stress concentration factor on the shoulder was about 1.18. As shown in Table 3, the radius increases with the decrease of the $K_{f}$.

**Table 3:** Stress concentration factor and arc radius.

Radius, mm	15	20	25	50	100
$K_{f}$	1.27	1.22	1.18	1.07	1.04

For material DP4, the total tensile force drops rapidly after the first Lüders band occurs on the stress concentration area. Work hardening is insufficient to support the stress to increase to a higher strength level and promote another Lüders band generated or moving forward. Garrell reported a solution to reduce the magnitude of stress concentration by increasing the radius of the arc in the transitional section [43]. This method cannot solve the earlier breaking problem of ultra-high strength DP steel after pre-strain and backing.

A rolling pin shape tensile specimen was developed to solve the earlier breaking on the shoulder. As shown in Fig. 7, the material was cut into a rolling pin shape specimen (RPS) in the first stage, stretched with a certain pre-strain, and baked with specific treatment parameters. Then the specimen of the first stage will be cut into a standard tensile specimen (STS) for second tensile testing. Hypothetically, the sum of the bake-hardening value (BH) and work-hardening value (WH) increases with the increase of pre-strain [44,45]. So, If the BH + WH of the stress concentration area is enough to prevent Lüders band from starting on the shoulder, the fracture will be transferred to the range of gauge length.

Three DP4 roll pin shape specimens were stretched with 2 % pre-strain. During the pre-strain process with the RPS, the stress rate of the modulus of elasticity testing was 30 MPa/s, the strain rate of yielding was 0.00025 $s^{-1}$, and the strain rate after yielding was 0.0067 $s^{-1}$. The specifications of tensile testing after baking were the same as STS. By this method, all of the DP4 specimens’ necking areas appeared on the parallel section after baking. As shown in Table 4, the upper yield strength is about 120 MPa higher than the tensile strength. The total elongation is about 1.2 %, reflecting a location deformation on the specimen.

**Table 4:** Mechanical properties of DP4 after pre-strain and backing tested by the RPS method.

Specimen No.	Upper yield stress, MPa	Lower yield stress, MPa	Tensile stress, MPa	Total elongation, %
1	1470	1351	1364	1.1
2	1491	1347	1361	1.1
3	1455	1345	1357	1.3

**Fig. 8:**
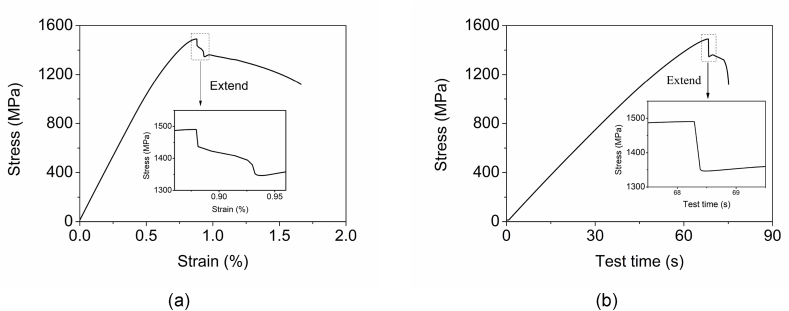
Tensile test curve and yielding partial enlargement curve of DP4 with rolling pin shape tensile specimens. (a) Stress and strain curve. (b) Stress and time curve.

As shown in Fig. 8, the upper yield stress is noticeably higher than the engineering tensile strength. A partial enlargement curve on the stress and strain curve shows that the stress drops from the top point to an oblique platform the first time and then drops to the lower yield point the second time. Nevertheless, no platform can be seen on the stress and time curve during dropping. The lower-yielding point should be the point at the bottom of the cliff. Interestingly, a dull sound like a breaking can be heard during the sudden stress drop after the upper yield point. This sound comes from the tensile force establishing and hitting on the clamping chuck of the tensile machine.

#### 3.1.3 DIC and FEA results of strain distribution

**Fig. 9:**
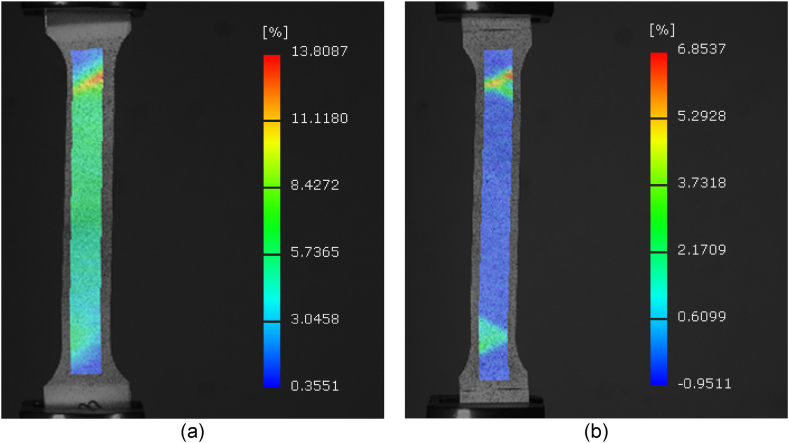
(a) DIC result of DP4 standard tensile test without pre-strain and baking. (b) DIC result of DP4 tensile test with 2 % pre-strain baked at 170 °C for 20 min.

As shown in Fig. 9 (a), during the standard tensile testing of DP4, the strain distribution is uneven, and there are smaller deformation areas near the outer side of transition sections. Even if the fracture appears near the transition section, the entire gauge length of the specimen has undergone significant uniform deformation. As shown in Fig. 9 (b), the strain distribution of DP4 during SBH testing was highly uneven, with deformation mainly concentrated in the transition sections at both ends, and no plastic deformation was detected within the gauge length.

**Fig. 10:**
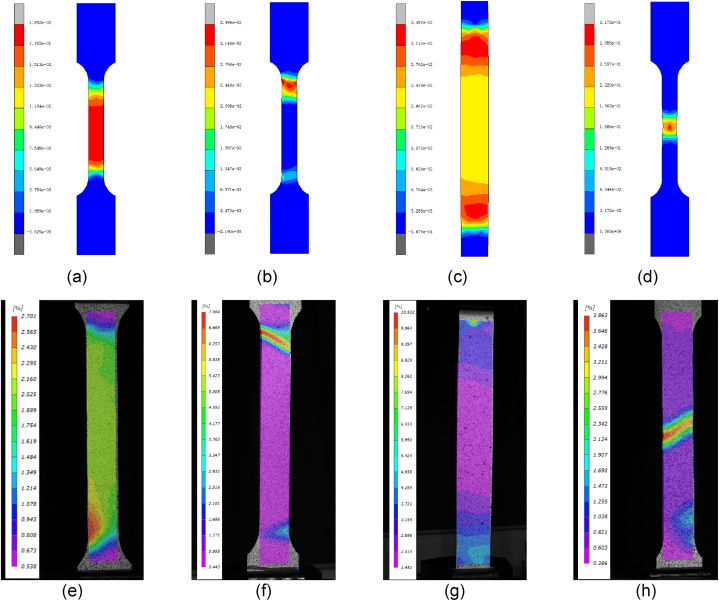
The FEA and DIC results of strain distribution comparison of DP4 between SBH and RPS. (a) SBH with 2 % pre-strain by FEA. (b) SBH after baking by FEA. (c) RPS with 2 % pre-strain by FEA. (d) RPS after baking by FEA. (e) SBH with 2 % pre-strain by DIC. (f) SBH after baking by DIC. (g) RPS with 2 % pre-strain by DIC. (h) RPS after baking by DIC.

The FEA and DIC results are compared in Fig. 10. The strain distribution results of both are almost identical. It means that the FEA can be used to analysis the fracture behavior of the specimens. Based on the correlation between pre-strain and aging strengthening, a higher strength at the location of the baked specimens with larger pre-strains was set. For the SBH method, as seen in Fig. 10 (a) and (e), the strain around the transition section is smaller than the gauge length section after the pre-strain. After baking, as seen in Fig. 10 (b) and (f), the gauge length section will have a higher strength, coupled with the stress concentration on the shoulder. The fracture position tends to be on the shoulder. For the RPS method as Fig. 10 (c) and (g), the strain near the transition section is larger than the gauge length section after the pre-strain. After baking, as seen in Fig. 10 (d) and (h), the gauge length section will have a lower strength. As a result, during the tensile test, the fracture position will be transferred to the gauge section after baking.

### 3.2 The strain aging behavior

#### 3.2.1 The strain aging behavior related to metallography

**Fig. 11:**
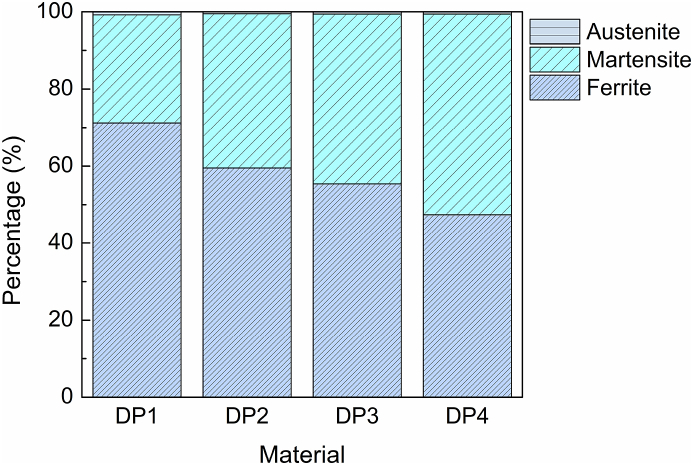
Phase percentage of different materials.

**Fig. 12:**
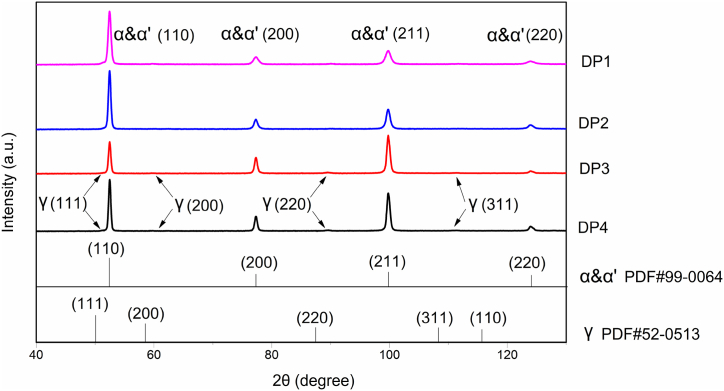
XRD result of materials.

Using the metallography method and XRD, the materials' ferrite, austenite, and martensite phase percentage was calculated and shown in Fig. 11. The results in Fig. 12 reveal the XRD patterns of materials at room temperature tested by Co Kα radiation. It shows the diffraction peaks of body-centered tetragonal martensite, ferrite, and face-centered austenite. But the signal of austenite is feeble. Using Jade software, the austenite percentages of all four materials were lower than 0.8 % as estimated. The diffraction peaks of austenite shift to the right side, which prefigures that the austenite has distortion.

**Fig. 13:**
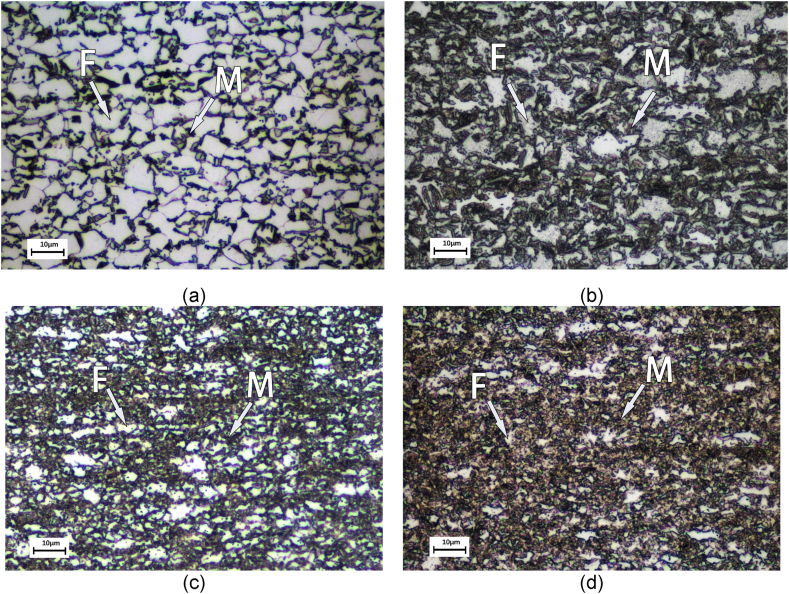
Metallography of materials, (a) DP1. (b) DP2. (c) DP3. (d) DP4.

The microstructures of materials mainly consist of a mixture of martensite and ferrite (Fig. 13). The percentages of martensite and ferrite were calculated by Image-Pro Plus on the metallographic photos after grayscale and contrast adjusting. The martensite phase percentage of DP4 is 52 %, which is observably higher than all other materials. Compared with the ferrite grain size, the average diameter of DP3 and DP4 is about 3.2 μm, which is finer than that of DP1 and DP2 (about 11.2 μm). So, suffering from the same pre-strain of 2 %, a higher dislocation density in the ferrite grain interior of DP3 and DP4 will be produced.

**Fig. 14:**
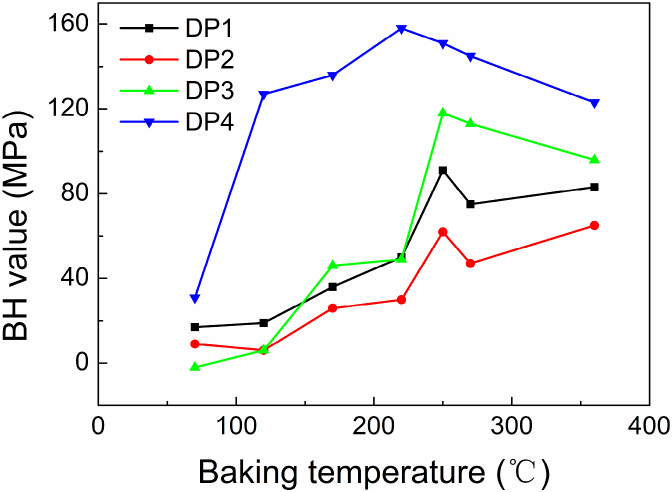
The BH value of materials baked at different temperatures after 20 min.

After 2 % pre-strain and baking from 70 °C to about 220 °C for 20 min, the BH value of the materials in research will be increased. When the baking temperature is higher than about 250 °C, generally, the BH value will be decreased (Fig. 14). Significantly, the BH value increasing rate of DP4 is considerably higher than other materials at the first stage. This may be related to the higher density dislocation in the lower proportion of ferrite in DP4. Because the distances between the dislocations and the interstitial atoms in DP4 are shorter, the energy required for the interstitial atoms to move towards the dislocations is smaller, and dislocations can quickly capture pinning atoms at a lower baking temperature.

#### 3.2.2 The strain aging behavior related to temperature

**Fig. 15:**
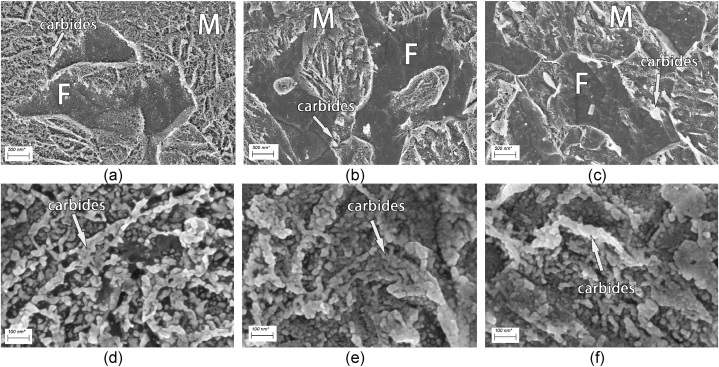
SEM results of DP4 with different baking temperatures: (a, d) quenched; (b, e) 170 °C baked for 60min; (c, f) 350 °C baked for 60min.

During the baking process, the martensite of the DP steel will be tempered. Research on DP steel shows that precipitation of carbides in the ferrite phase, martensite, and ferrite/martensite interfaces can be observed after tempering [46,47]. Compared to quenched, coarsening of carbide precipitates can be seen after baking at 170 °C for 60min (Fig. 15 (a) and (b)). Coarser carbides were produced after baking at 350 °C for 60min (Fig. 15 (c)).

**Fig. 16:**
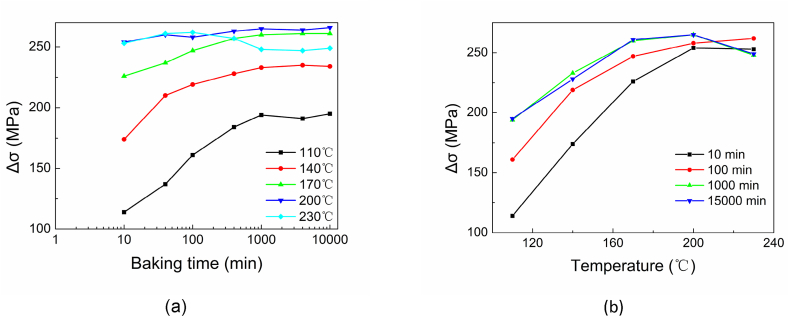
Strain aging of DP4 with different baking temperatures.

Fig. 16 (a) describes the behavior of DP4 of changes in yield stress Δσ with 2 % pre-strain at different baking temperatures. The Δσ increases with the increase of baking time and reaches an almost constant platform. Baking temperature from 110 °C to about 200 °C shows that a higher baking temperature results in a higher maximum platform exhibited by DP4. In the case of baked with 230 °C, the Δσ increases at the first stage and reaches a peak in 100 min. Subsequently, the Δσ decreases after it reaches the constant platform. Again, the Δσ remains at a lower constant platform after 1000min.

When baking time is longer than 1000 min, the curves of Δσ plotted in Fig. 16 (b) almost overlap. There are Δσ peaks around 200 °C could be found on this cluster of curves. It can be inferred that baked with 230 °C, the Δσ decreasing after peak could be mainly due to the depinning of dislocation and precipitation of carbides, not reversion of dislocation. Evidence shows the edge dislocations, which form the Cottrell atmosphere, are not eliminated until 350 °C [48]. For commercial iron, when the tempering temperature is higher than 400 °C, an apparent dislocation recovery is detected by positron annihilation spectroscopy [49]. Even annealed at 227 °C for 10 h, the decrease in steel dislocation density will not be noticeable [50].

#### 3.2.3 The strain aging behavior related to pre-strain

**Fig. 17:**
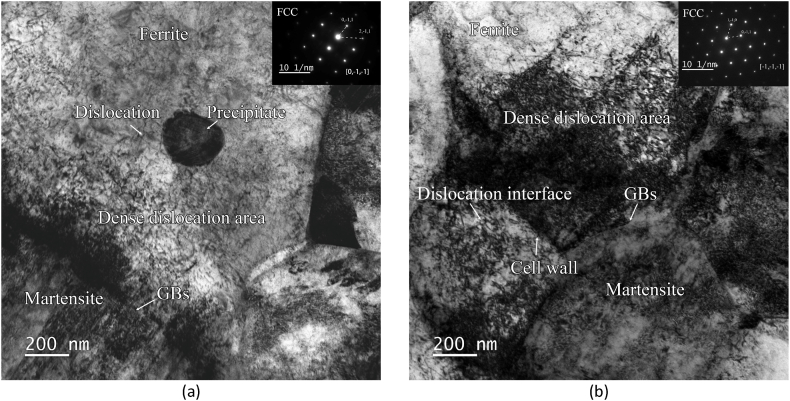
TEM result of DP4 with different pre-strain: (a) without pre-strain; (b) 2 % pre-strain.

Fig. 17 (a) shows the TEM result of DP4 without pre-strain. The dense dislocation areas around the martensite and ferrite interface can be seen on the ferrite side. A large number of dislocations also display around the precipitates. As Fig. 17 (b) shows, when a 2 % pre-strain deformation is applied to DP4, the dislocation density increases inside the ferrite. The dislocation cells were produced. These dislocations provide an essential place for the formation of the Cottrell atmosphere.

**Fig. 18:**
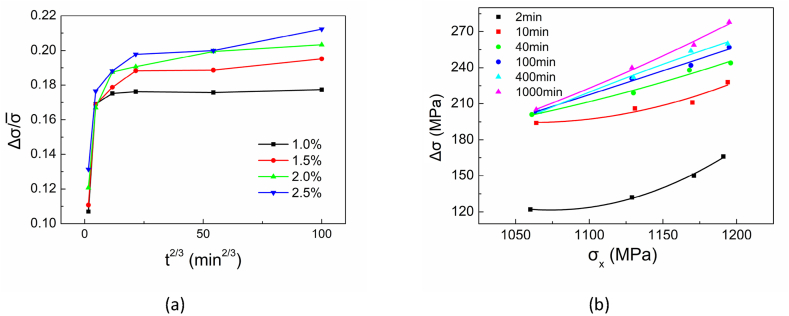
Strain aging of DP4 with different pre-strains baked at 170 °C.

For the DP4 material deformed with different pre-strains and then baked at the same temperature of 170 °C, the strain aging behavior section can be regarded as three stages in Fig. 18 (a).

(1) The strain aging with different pre-strain obeys the relationship modified by Hartley from Cottrell–Bilby at the beginning of strain aging.
(2) The slope S of Eq. (2) will be reduced. Then the yield strength increases to a stress platform.
(3) When the aging time is long enough, the yield strength remains constant at the last stage. The platform value increases with the increase of the pre-strain in a particular range.

When baked for 2–40 min, a higher pre-strain corresponds to a higher increasing ratio $d\Delta \sigma /{d\sigma }_{x}$ as shown in Fig. 18 (b). Approximately, when the baking time is longer than 40 min, Δσ and $\sigma_{x}$ are linear. Baked for more than 1000 min, the total available dislocations which can be calculated by ${\rho_{total}}^{*}=\left({\rho_{m}}^{*}+{\rho_{f}}^{*}\right)$ are almost immobile, or ${\rho_{total}}^{*}\approx {\rho_{f}}^{*}$. The strengthening fraction $\left(1-{\rho_{f}}^{*}/\left({\rho_{m}}^{*}+{\rho_{f}}^{*}\right)\right)$ of $\sigma_{ss}$, $\sigma_{p}$ and $\sigma_{GB}$ in Eq. (4) will approach zero. Therefore, it can be inferred that pinning dislocation density and forest dislocation density have the same contribution coefficient to strength.

#### 3.2.4 Internal friction analysis

Before internal friction testing, the DP4 after 2 % pre-strain was baked at 170 °C for 0, 5, 20, and 120 min respectively. The DP1 after 2 % pre-strain baked with 170 °C for 20 min was researched for comparison. To fit the spectrum and calculate the height of peaks, the background was subtracted according to the exponential function [51]:

(6) $$Q_{b}^{-1}=A+Be^{\left(\frac{c}{k_{B}T}\right)}$$

where A, B, and C are the parameters determined after optimization of the $\chi^{2}$ function; $k_{B}$ is the Boltzmann constant; T is the temperature.

**Fig. 19:**
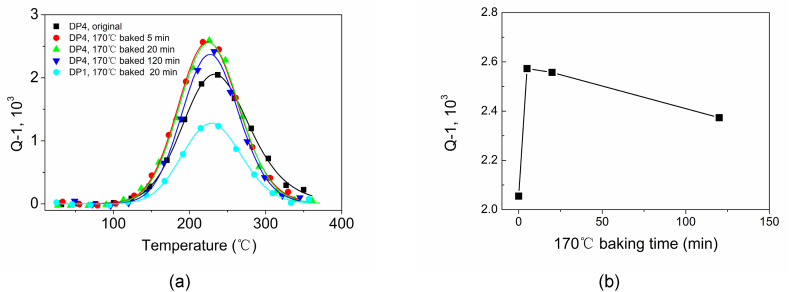
Damping properties of dual phase steel DP4 and DP1. (a) Temperature-dependent variation of the damping capacity of different baking times at 170 °C. (b) The relation between the height of the SKK peak of damping and the baking time of DP4.

A weak Snoek peak of the DP4 baked lower than 5 min at 170 °C can be observed around 67 °C in Fig. 19 (a). As Snoek peaks were caused by interstitial atoms in a body-centered cubic [52], it can be conjectured that the interstitial atoms in the iron lattice could diffuse to the dislocation during over aging just after fast cooling. During baking, the interstitial atoms were removed from the lattice of higher dislocation density, so the Snoek peak almost disappeared quickly. On the other hand, the Snoek-Kê-Kster (SKK) peak of DP4 around 227 °C increases with increasing baking time. When the baking time is higher than 20 min, the rise of the SKK peak almost decreases in Fig. 19 (b). The internal friction due to dislocation pinning corresponds to the law of strain aging measured with Δσ as seen in the above presentation, proving that stain aging strongly correlates with dislocation pinning [53]. When the baking time is longer than 120min, the SKK peak decreases. It indicates the presence of depinning and carbide precipitation. With the same process of baking at 170 °C for 20 min after 2 % pre-strain, the SKK peak of DP4 becomes twice as high as that of DP1. This shows that the interaction between interstitial atoms and the dislocation of DP4 is more intense than DP1.

## 4 Discussion

### 4.1 The dislocation pinning quantization

In the first stage, in the beginning, the strain aging behavior obeys Cottrell–Bilby's theory very well. The free dislocations are sufficient to provide positions for pinning by interstitial atoms, showing quite a linear dependence on the degree of atmosphere formation [23]. Nevertheless, as the free dislocation density decreases and the interstitial atoms close to free dislocation are consumed, relying on Cottrell–Bilby theory alone will not support a higher pinning rate. Therefore, the pinning rate will be limited by a statistical probability.

Hypothetically, dislocation pinning intensity is quantized. It means the total number of mobile dislocations at the beginning of the second stage are known in a lower aging temperature without depinning and dislocation recovery. In that way, the total positions on the dislocations able to be used for pinning are known as $N_{0}$. At the same time, it assumes that the diffusion coefficient of interstitial atoms is a constant. The interstitial atoms and free dislocation distribution are uniform in the lattice. The probability of interstitial atoms diffusing to each free dislocation is almost a constant p. Then, the number of residual free dislocation pinning positions $N_{m}$ for each time can be expressed as:

(7) $$\begin{array}{l} N_{m}\left(t_{0}\right)=N_{0}\\ N_{m}\left(t_{1}\right)=N_{0}-p_{1}\cdot N_{0}\\ N_{m}\left(t_{2}\right)=N_{m}\left(t_{1}\right)-p_{2}\cdot N_{m}\left(t_{1}\right)\\ \cdots \\ N_{m}\left(t_{n}\right)=N_{m}\left(t_{n-1}\right)-p_{n}\cdot N_{m}\left(t_{n-1}\right) \end{array}$$

where the $p_{n}$ is the probability at time $t_{n}$. Then the residual free dislocation pinning positions $N_{m}$ becomes:

(8) $$N_{m}\left(t_{n}\right)=N_{0}\cdot \prod_{i=1}^{n}\left(1-p_{i}\right)$$

As the pinning rate at the first stage is higher than calculated from probability p, hence, a timely constant a should be taken into consideration. Finally, after the aging time, it will be:

(9) $$N_{p}=N_{0}-N_{0}\cdot {\left(1-{\bar{p}}_{p}\right)}^{t+a}$$

where $N_{p}$ is the pinned positions of dislocations after time t; ${\bar{p}}_{p}$ is the average probability for pinning.

In practical terms, the probability p is a dependent variable affected by many factors, such as interstitial atom density, dislocation density, diffusion coefficient, category of interstitial atoms, precipitation of carbides, etc. Fig. 16 (b) shows a higher aging temperature till about 200 °C related to higher yield stress Δσ. This may be caused by higher potential energy positions being opened, the available positions for pinning being increased, and a larger $N_{0}$. Replacing the pinned dislocation positions into pinned dislocation density. Based on Taylor hardening law Eq. (3), introducing the above probability inference, the increased yield stress $\Delta \sigma_{p}$ by pinning can be improved as:

(10) $$\Delta \sigma_{p}=M_{T}\alpha \mu b\sqrt{\rho_{p}}=M_{T}\alpha \mu b\sqrt{\rho_{0}-\rho_{0}\cdot {\left(1-{\bar{p}}_{p}\right)}^{t+a}}$$

where $\rho_{p}$ is pinned dislocation density; $\rho_{0}$ is the total dislocation density that can be used for pinning. During a higher aging temperature, for example, 230 °C, a higher depinning and precipitation rate will break the previous balance, and cause decreasing in yielding stress Δσ. Also, it assumes that there are no dislocations recovery during the aging below 300 °C. In this case, the depinning of dislocation is also a probability event, so the depinning under the dislocation can be expressed:

(11) $$N_{d}=n_{0}-n_{0}\cdot {\left(1-{\bar{p}}_{d}\right)}^{t+b}$$

where $N_{d}$ is the depinned dislocation positions after time t; $n_{0}$ is the total dislocation positions that can be used for depinning after long time aging; ${\bar{p}}_{d}$ is an average probability of interstitial atoms depinning and precipitated subsequently; t is the aging time; b is a correction constant. Therefore, if the pinning and depinning are in progress at the same time to keep a balanced status, the final yield stress changing Δσ can be further expanded to:

(12) $$\Delta \sigma =M_{T}\alpha \mu b\sqrt{\rho_{0}-\rho_{d}-\rho_{0}\cdot {\left(1-{\bar{p}}_{p}\right)}^{t+a}+\rho_{d}\cdot {\left(1-{\bar{p}}_{d}\right)}^{t+b}}$$

where $\rho_{d}$ is the total dislocation density that can be used for depinning after a long aging time.

**Fig. 20:**
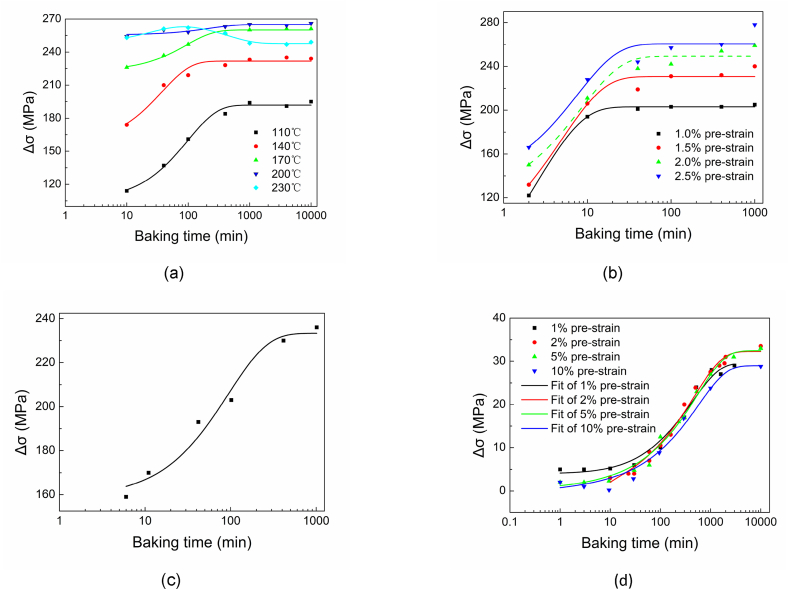
Comparisons of experimental and fitting results. (a) 2 % pre-strain with a different aging temperature of DP4; (b) 170 °C aging with different pre-strain of DP4; (c) 2 % pre-strain with 170 °C aging of DP3; (d) 50 °C aging with different pre-strain of ultra-low carbon bake hardening steel in Ref. [23].

For a better practicability verification of the results, Fig. 20 shows the comparison between experimental results and fitting results. Whether DP4 after 2 % pre-strain baked in the different temperatures from 110 °C to 200 °C, or after different pre-strains baked at 170 °C, the curves fitted by Eq. (10) agree with the observation data. Some points at the end of a plateau in Fig. 20 (b) distribute above the fitted curves, it suggested that it’s due to the precipitation of carbides. It's increasing to the second plateau. The result of DP4 after 2 % pre-strain baked in 230 °C will meet the fitted result of Eq. (12) in Fig. 20 (a). The aging result of DP3 also can be fitted well with equations as seen in Fig. 20 (c). The aging effects of ultra-low carbon bake hardening steel reported in A.K. De's research [23] are also fitted in Fig. 20 (d). Overall, these experimental results can be well fitted with Eq. (10), except for a few points at the very beginning of the aging stage distributed above curves in Fig. 20 (d), which could be resulted in a higher aging rate following Cottrell–Bilby theory as discussed above.

Regarding the strengthening law of Eq. (4), the $\rho_{f}$ can be split at least into two types of immobile dislocation densities, which are pinning dislocation density $\rho_{p}$ and forest dislocation density $\rho_{r}$. Therefore, the strengthening model of yield stress $\sigma_{y}$ proposed by Jiang [38] can be improved as below:

(13) $$\sigma_{y}=\sigma_{0}+\left(\sigma_{ss}+\sigma_{p}+\sigma_{GB}\right)\left(1-\frac{\rho_{p}+\rho_{r}}{\rho_{m}+\rho_{p}+\rho_{r}}\right)+M_{T}\alpha \mu b\sqrt{\rho_{p}+\rho_{r}}$$

where $\rho_{f}$ is the total immobile dislocation density, and $\rho_{f}=\rho_{p}{+\rho }_{r}$. Therefore, a longer baking time of the material, a lower percentage of the $\rho_{m}$. As a result, the impact of the $\left(\sigma_{ss}+\sigma_{p}+\sigma_{GB}\right)$ item is less.

### 4.2 Fracture of tensile specimen

**Fig. 21:**
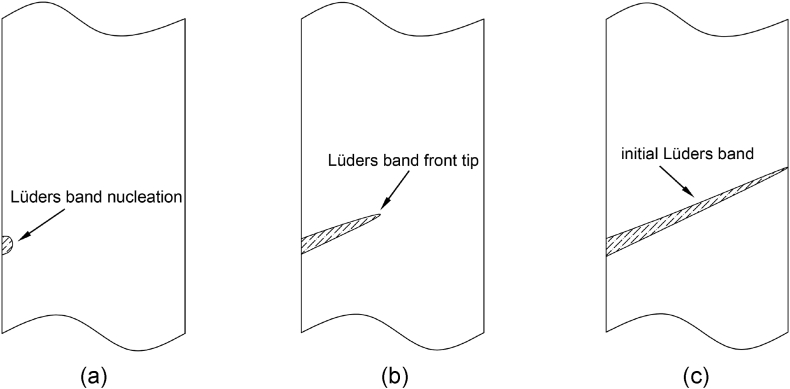
Initial Lüders band formation.

As shown in Fig. 21, suppose that the initial Lüders front nucleated at the point of the edge of the specimen shoulder. Then the Lüders front tip will be propagated across the specimen section quickly [54], leading to an initial band formation and stress avalanche.

The Lüders front inclinations φ to the tensile axis of an SBH test for DP4 are always in the range of 59°–64°. Using the analytical results of Rainer Schwab [55], the approximate true lower-stress and true upper-stress values could be about 1086 MPa and 1955 MPa, respectively, which are different from the stress observed on the tensile curve.

**Fig. 22:**
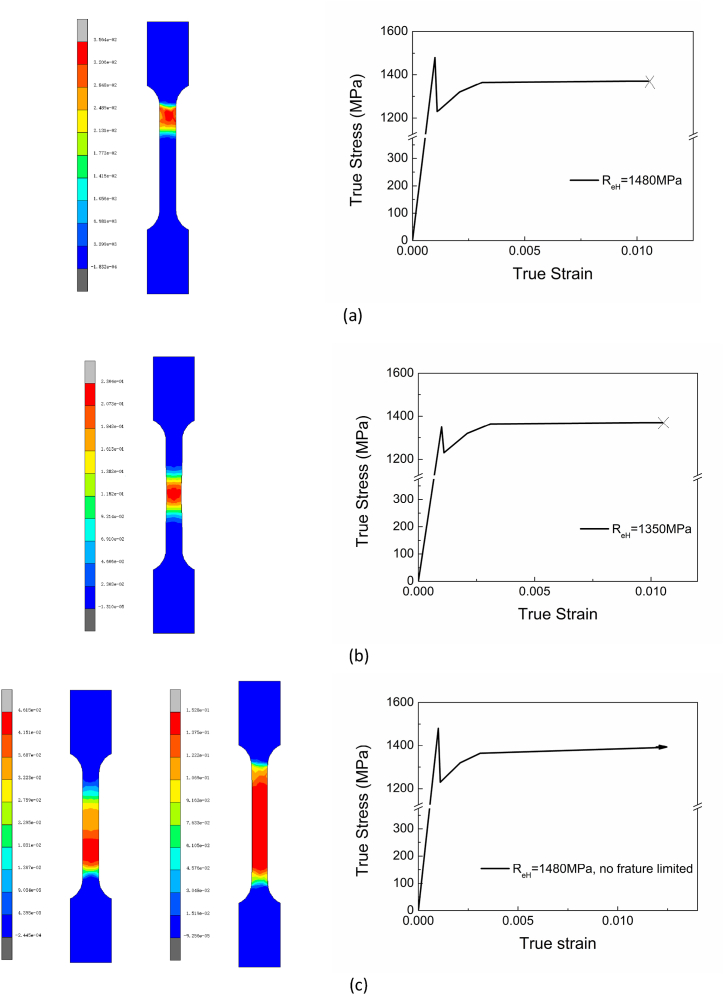
Fracture positions of different upper yield strengths with the same lower yield stress 1230 MPa. (a) Upper yield stress is 1480 MPa, and stress at fracture is 1370 MPa. (b) Upper yield stress is 1350 MPa, and stress at fracture is 1370 MPa. (c) Upper yield stress is 1480 MPa, without fracture limited.

The FEM of homogeneous materials with different upper yield strengths was conducted. As shown in Fig. 22 (a), assuming that the upper yield stress is greater than the stress at fracture, the specimen will fracture near the transition section. For Fig. 22 (b), if the upper yield stress is less than the stress at fracture, the fracture tends to locate in the gauge distance. But suppose there is a positive harden index always existing after yielding in the case of Fig. 22 (c), which means that without fracture limit, the strain will firstly occur around the transition section and then expand to the entire gauge length. It indicates that the upper yield strength and total elongation impact the fracture's location, or the higher upper yield strength and lower total elongation will promote local deformation.

Compared to DP1 and DP2, only a smaller part of the refined ferrite grains in DP4 undertake most plastic deformation. Then the stress contributed by dislocation density in Eq. (13) of DP4 will be much higher. Due to a higher percentage of dislocations being pinned after strain aging, the upper yield strength of DP4 will be higher. Also, the average travel distance of dislocations is much shorter to make a dislocation interaction, leading to localized deformation [56]. In the Lüders band area of DP4, although the forest dislocation density $\rho_{r}$ increase with increased deformation in the grain interior, as the sectional area decreases at the same time, critical flow stress is not reached the ultra-high upper stress controlled by the dislocation pinning on the elastic side for triggering a Lüders band propagation. Additionally, corresponding to the inhomogeneous pre-strain, the upper yield stress inside the gauge length of an SBH specimen was higher than the transition section, so the necking and fracture on the specimen shoulder will occur.

## 5 Conclusion

In this paper, the reason for fracture outside gauge length of a tensile specimen of bake hardening value test was analyzed, the effects of different microstructure, pre-strain, baking temperature, and baking time on the strain aging of dual phase steels were studied, and the strengthening mechanism of dislocation pining during saturation state was investigated. The conclusions are summarized as follows.

(1) When using SBH specimens to test the BH value of ultra-high strength dual phase steels, the fracture outside the gauge length is mainly related to the uneven distribution of pre-strain and ultra-high upper yield strength. After baking, the upper yield strength in the gauge length is significantly higher than that in the transition section, and the stress concentration in the transition section promotes preferential yielding in this section. The fracture stress of the transition section is significantly lower than the upper yield strength of the gauge length, making it difficult to extend the deformation to the gauge length, resulting in a continuous reduction of the cross-sectional area of the transition section and further fracture.
(2) The testing method for rolling pin shape tensile specimen introduced in this paper utilizes the correlation between pre-strain and bake hardening of ultra-high strength dual phase steel, improves the strength of the transition section of the baked specimen, avoids yielding and deformation in this section, and promotes deformation and fracture in the gauge length section.
(3) The ultra-high upper yield strength of dual-phase steel after strain aging is related to the high density of dislocations in ferrites and their pinning. During baking, high-density dislocations are quickly pinned by interstitial atoms, resulting in greater energy required for dislocation migration in the ferrites after baking, thus leading to a higher upper yield stress.
(4) The proposed dislocation pinning strengthening mechanism is based on quantization assumption and probability event. When dislocation pinning develops to the saturation stage, the pinning rate gradually deviates from the Cottrell-Bilby theory. But the fitting results based on the quantization mechanism agree with the measured results at the saturation stage.

## Ethics approval and consent to participate

Not applicable.

## Data availability statement

Data will be made available on request.

## Additional information

No additional information is available for this paper.

## Author contribution statement

Biao Xiao: Conceived and designed the experiments; Performed the experiments; Analyzed and interpreted the data; Contributed reagents, materials, analysis tools or data; Wrote the paper. Jie Zhou: Analyzed and interpreted the data. Jean-Luc Christen: Analyzed and interpreted the data. Weimin Zeng: Performed the experiments. Wenyi Peng: Analyzed and interpreted the data. Wrote the paper.

## Declaration of competing interest

The authors declare the following financial interests/personal relationships which may be considered as potential competing interests: Xiao Biao has patent #ZL2020105646203 and ZL2019111451661 licensed to Valin ArcelorMittal Automotive Steel Co., LTD.
